# Minimal influence of estrous cycle on studies of female mouse behaviors

**DOI:** 10.3389/fnmol.2023.1146109

**Published:** 2023-07-04

**Authors:** Pei-Yun Zeng, Ya-Hsuan Tsai, Chih-Lin Lee, Yu-Kai Ma, Tsung-Han Kuo

**Affiliations:** ^1^Institute of Systems Neuroscience, National Tsing Hua University, Hsinchu, Taiwan; ^2^Department of Life Science, National Tsing Hua University, Hsinchu, Taiwan; ^3^Brain Research Center, National Tsing Hua University, Hsinchu, Taiwan

**Keywords:** estrous cycle, behavioral variation, sex bias, female mouse behaviors, sex differences

## Abstract

**Introduction:**

Sex bias has been an issue in many biomedical fields, especially in neuroscience. In rodent research, many scientists only focused on male animals due to the belief that female estrous cycle gives rise to unacceptable, high levels of variance in the experiments. However, even though female sexual behaviors are well known to be regulated by estrous cycle, which effects on other non-sexual behaviors were not always consistent in previous reports. Recent reviews analyzing published literature even suggested that there is no evidence for larger variation in female than male in several phenotypes.

**Methods:**

To further investigate the impact of estrous cycle on the variability of female behaviors, we conducted multiple behavioral assays, including the open field test, forced swimming test, and resident-intruder assay to assess anxiety-, depression-like behaviors, as well as social interaction respectively. We compared females in the estrus and diestrus stages across four different mouse strains: C57BL/6, BALB/c, C3H, and DBA/2.

**Results:**

Our results found no significant difference in most behavioral parameters between females in these two stages. On the other hand, the differences in behaviors among certain strains are relatively consistent in both stages, suggesting a very minimal effect of estrous cycle for detecting the behavioral difference. Last, we compared the behavioral variation between male and female and found very similar variations in most behaviors between the two sexes.

**Discussion:**

While our study successfully identified behavioral differences among strains and between the sexes, we did not find solid evidence to support the notion that female behaviors are influenced by the estrous cycle. Additionally, we observed similar levels of behavioral variability between males and females. Female mice, therefore, have no reason to be excluded in future behavioral research.

## Introduction

Experimental designs with sex bias have been recognized as an issue in both basic and translational research (Kong et al., 2016; Stephenson et al., 2018; Villavisanis et al., 2018; Xiao et al., 2018; Coiro and Pollak, 2019; Plevkova et al., 2020; Flynn et al., 2021; Mercel et al., 2021; Mondini Trissino da Lodi et al., 2022; Spitschan et al., 2022). Even in clinical studies, the number of female participants is underrepresented substantially (Mansukhani et al., 2016; Villavisanis et al., 2018; Feldman et al., 2019; Karp and Reavey, 2019; Burra et al., 2022; Mondini Trissino da Lodi et al., 2022). Sex or gender differences, however, exist not only in reproductive organs but also in several other characteristics, including metabolism, disease patterns and drug pharmacokinetics. The treatments based on the studies of male participants may cause unanticipated or even adverse effects in female patients. Therefore, studying both sexes equally is important for minimizing poor replicability or translation failure in biomedical research.

The issue of sex bias also exists in animal research, especially in neuroscience. Approximately, for every 5.5 studied male animals, only one female animal was examined in neurosciences studies (Beery and Zucker, 2011). Rodents, including rats and mice, are the most common model organisms for biological research. A statistical study showed that, from 2010 to 2014, there were ~ 40% of papers in neuroscience using only males as experimental subjects, whereas the number of papers using only females remained at a constant low value, about 5% (Will et al., 2017). Like humans, male and female rodents are also different in morphology, physiology and behaviors. Given the significant impact of sex differences, in 2016, the National Institutes of Health implemented a guideline to consider sex as a biological variable and encouraged the use of both sexes in animal research. Despite that, female rodents were still excluded in a lot of behavioral research (Chen et al., 2018; Lin et al., 2018; Chevalier et al., 2020; Pati et al., 2020; Zhang et al., 2020; Felippe et al., 2021; Mahadevia et al., 2021; Wu et al., 2021; Zhang et al., 2022). For example, 44.5% of the papers focusing on maternal immune activation contained only behavioral data of male offspring, whereas 3.4% of studies only looked at female offspring (Coiro and Pollak, 2019). This ignorance of female mice in the experimental designs is probably due to the belief that estrous cycle gives rise to unacceptable, high levels of variance in female rodents.

Similar to the human menstrual cycle (Hawkins and Matzuk, 2008), the estrous cycle in female rodents presents a cyclic change in ovulation regulated by reproductive hormones. It can be divided into four stages, proestrus, estrus, metestrus and diestrus and typically lasts 4 to 5 days for rats and mice (Ajayi and Akhigbe, 2020). During this cycle, the sex hormones undergo dramatic out-of-phase fluctuations in the level of secretion. In the metestrus and diestrus stages, female rodents have low levels of estradiol. When female rodents are in the late proestrus stages, the elevated estradiol level induces the luteinizing hormone (LH) and Follicle Stimulating Hormone (FSH), which initiates ovulation during the estrus stage. The level of estradiol reaches peak concentration near the onset of estrus and drops rapidly after ovulation. The regulation of sexual behaviors by estrous cycle has been well established. During the estrus stage, females not only release pheromones to attract males but also showed more interest in male odors, more lordosis and higher sexual receptivity (Dey et al., 2015; Jennings and De Lecea, 2020).

Because these hormones affect female physiology significantly, many researchers believe that the estrous cycle can also influence other non-sexual behaviors, including emotion-related behaviors, drug taking, and cognition. The influence of estrous cycle on anxiety- and depression-like behaviors have been reported but the results were not always consistent (Lovick and Zangrossi, 2021). For example, some suggested that female mice showed more anxiety-like behaviors during the diestrus stage (Gangitano et al., 2009; Koonce et al., 2012; Rocks et al., 2022), but others showed there is no significant influence (Chari et al., 2020; Zhao et al., 2021; Francois et al., 2022). The effect of estrous cycle on social motivation based on three chamber social test of female mice was also observed in some reports (Chari et al., 2020), but not by others (Zhao et al., 2021). While these controversial results might be caused by a variety of confounding effects, such as genetic background, housing environment, or experimental setup (Palanza et al., 2001; Meziane et al., 2007; Kokras et al., 2015; Dossat et al., 2018; Miller et al., 2021), the inconsistency implied that the influence of estrous cycle on these behaviors might not be so substantial.

The assumption that female non-sexual behaviors are more variable than male’s was also being questioned recently. A review article re-analyzed data from published papers and provided a rebuttal to the conjecture that females are more variable than males (Prendergast et al., 2014). Direct comparison of variations in anxiety-like behavior, locomotive activity, feeding behavior, gene expression, dendritic spine density in ventral hippocampus between males and females also failed to reveal larger variation in females caused by the estrous cycle (Francois et al., 2022; Rocks et al., 2022; Smarr and Kriegsfeld, 2022). A new study using machine-learning software even showed that open-field exploration in males are much more variable than females (Levy et al., 2023).

Taken together, while female mice were often understudied in behavioral research due to the variation caused by the estrous cycle, whether females really showed larger variation than males remains an uncertain assumption. To validate this hypothesis, in this study, we applied four different inbred strains of female mice, C57BL/6, BALB/c, C3H, and DBA/2 to examine multiple non-sexual behaviors, including anxiety-like, depression-like behaviors and the same-sex interaction, between females in estrus and diestrus stages to test if the estrous cycle has an impact on any of these behaviors. The variation of these behaviors between C57BL/6 female and male mice was also compared to ask if the female mouse behavioral variation was greater than males. We are hoping that this study would help us clarify the exact influence of the estrous cycle on female mouse behaviors. The information could be an important guidance for future experimental design in behavioral neuroscience.

## Method

### Mice

C57BL/6 J, BALB/cByJ 7-week-old female mice and C57BL/6 J 7-week-old male mice were purchased from the National Laboratory Animal Center, Taiwan. C3H/HeNCrlBltw and DBA/2NCrlBltw 7-week-old female mice were purchased from BioLASCO Taiwan Co., Ltd., Taiwan. Mice were singly-housed in a controlled animal room with a 12-h light/dark cycle (0700–1900 h). Mice between the age of 8–10 weeks were used for behavioral tests during the light period. For habituation, mice were moved to the behavioral room for 1 hour at least 2 days before the experiments. At the experimental day, there was also one-hour habituation before the 1st assay. Open field test and forced swimming test were conducted under the light condition (40 W). Resident-intruder assay were conducted under the red-light environment (5 W). All animal procedures were in compliance with institutional guidelines established and approved by the Institutional Animal Care and Use Committee of National Tsing Hua University.

### Experimental procedure

**Figure 1:**
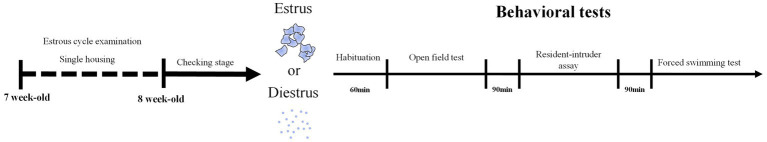
Experiment Flowchart. At 7-week-old, mice were single-housed and examined for the estrous cycle stage for a week. At 8-week-old, estrus and diestrus female mice went through the open-field test, resident-intruder assay followed by the forced swimming test.

Upon mice arriving, we singly-housed the mice and used the microscope to examine the estrous cycle stage every day for a week (Figure 1). After validating that their estrous cycles are normal, we continued to check the stages and chose females in diestrus or estrus stage to conduct the open field test, resident-intruder assay, and forced swimming test within a day (Figure 1). Therefore, each mouse was tested in two trials, in estrous and diestrus stages, for all three assays following the same order. There was no cage change during the experimental period.

### Ovariectomized female intruders

BALB/cByJ females were used as intruders for female resident-intruder assay. Mice were ovariectomized under anesthetization (Olson and Bruce, 1986). Hairs on the back were shaved off for surgery. The skin and muscles 0.5 cm beneath the midline of the back were incised. The fat beneath the muscles was grasped to exteriorize the ovary. Then the ovary was removed by cutting. Finally, the incisions in the muscle and skin were closed.

### Non-aggressive male intruders

BALB/cByJ males were used as intruders for male resident-intruder assay. Intraperitoneal injection of 2,6-dichlorobenzonitrile (dichlobenil) has been shown to ablate olfactory neuroepithelium and minimize aggression (Brandt et al., 1990; Ma et al., 2022). Mice were intraperitoneally injected with 50 μL dichlobenil (50 mg/mL) every other day for three times. Next day after the last injection, mice can be used as intruders for the assay.

### Estrous cycle examination

Female estrous stage was identified by vaginal cells. Mice tail is elevated to visualize the vagina. The female vaginal contents were collected by placing a small drop of PBS into the vaginal and washing for 4 to 5 times by pipetman. The vaginal cell suspension was then immediately observed under a microscope. The phases of the estrous cycle were evaluated based on the differences in cellular shape (Ajayi and Akhigbe, 2020). In the estrus stage, the cells are most irregular in shape, and the diestrus stage shows prominent polymorphonuclear leukocytes. Because proestrus and metaestrus are more difficult to be identified and therefore less reliable for study (Walf and Frye, 2006; Becker and Hu, 2008; Walf et al., 2009), we followed most studies to only focus on estrus and diestrus stage (Caligioni, 2009; Kokras and Dalla, 2014; Becker and Koob, 2016; Numan and Young, 2016). The stage was examined every morning for a week to validate the normal estrous cycle before the experiment.

### Open field test

The open field test was used to evaluate locomotion activity and anxiety-like behavior in rodents (Seibenhener and Wooten, 2015). A mouse was placed into the center of a clean 50 cm × 50 cm open field apparatus for 10 min with video recording overhead. SMART VIDEO TRACKING Software (Panlab) was used to determine the total moving distance and total time in the box center (25 cm × 25 cm). The activity was presented by the total moving distance. The anxiety level was presented by the time mice spent, number of entries and distance in the box center.

### Standard forced swimming test

The forced swimming test was applied to study rodent’s depressive-like behavior (Can et al., 2012; Chen et al., 2015). A mouse was placed into the container [11.5 cm (R) × 17.5 cm (H)], which was half filled with water to prevent the mouse from stepping on the bottom or escaping from the top. The behavior was recorded immediately by the digital camera. After six minutes, mice were removed from the water and dried with an infrared lamp in their homecage. We used SMART VIDEO TRACKING Software (Panlab) to analyze the last four minutes video to avoid unstable immobility behavior in the first 2 min (Can et al., 2012; Chen et al., 2015). Immobility was characterized by floating or minimal movement for maintaining balance in the water.

### Resident-intruder assay

The resident-intruder assay was applied to investigate mouse social motivation (Ma et al., 2022). Ovariectomized BALB/cByJ females and non-aggressive BALB/cByJ males were used as intruders. Intruders were introduced into the homecage (18.1 cm × 39.8 cm) of the subject mice for 10 min. Social interaction was recorded with a video camera. The videos were then analyzed manually using Behavioral Observation Research Interactive Software (BORIS) to obtain the total time for social interaction, including aggression, allogrooming and general social investigation. Number of social bouts, latency to approach and self-grooming time were also analyzed. Intruders were returned to their homecage after each test.

### Statistics

All statistics were completed using GraphPad Prism 8.0.2 and SPSS 22.0 software. The Shapiro-Wilkinson normality test was used to analyze the distribution of the data. For the comparisons between the estrus and diestrus female, paired *t*-test was applied for normally distributed data, and the Wilcoxon matched-pairs signed-rank test was applied for non-normally distributed data. Multiple comparisons for different strains were examined by the Kruskal-Wallis test followed by the *post hoc* Dunn’s multiple comparisons test. To account for the trial effect, the Generalized Linear Mixed Model (GLMM) was utilized to examine differences between the two stages, with strain considered as random effect, or among strains, with stage considered as random effect. For the comparisons between the sexes, Welch’s t-test was applied for normally distributed data, and Mann–Whitney *U* test was applied for non-normally distributed data. The comparisons of variation between the sexes were examined by Standard deviation (SD), Coefficient of variation (CoV), F-test and Levene test. All data are represented as mean +/− standard error of the mean (S.E.M.).

## Result

### The estrous cycle has no significant influence on female mouse behaviors

To investigate the influence of estrous cycles on female mouse behaviors, we tested behaviors in four different inbred strains, C57BL/6, Balb/c, C3H, and DBA2. We first used a microscope to examine the estrous cycle stage, then performed the open field test, forced swimming test, and resident-intruder assay for both diestrus and estrus females.

**Figure 2:**
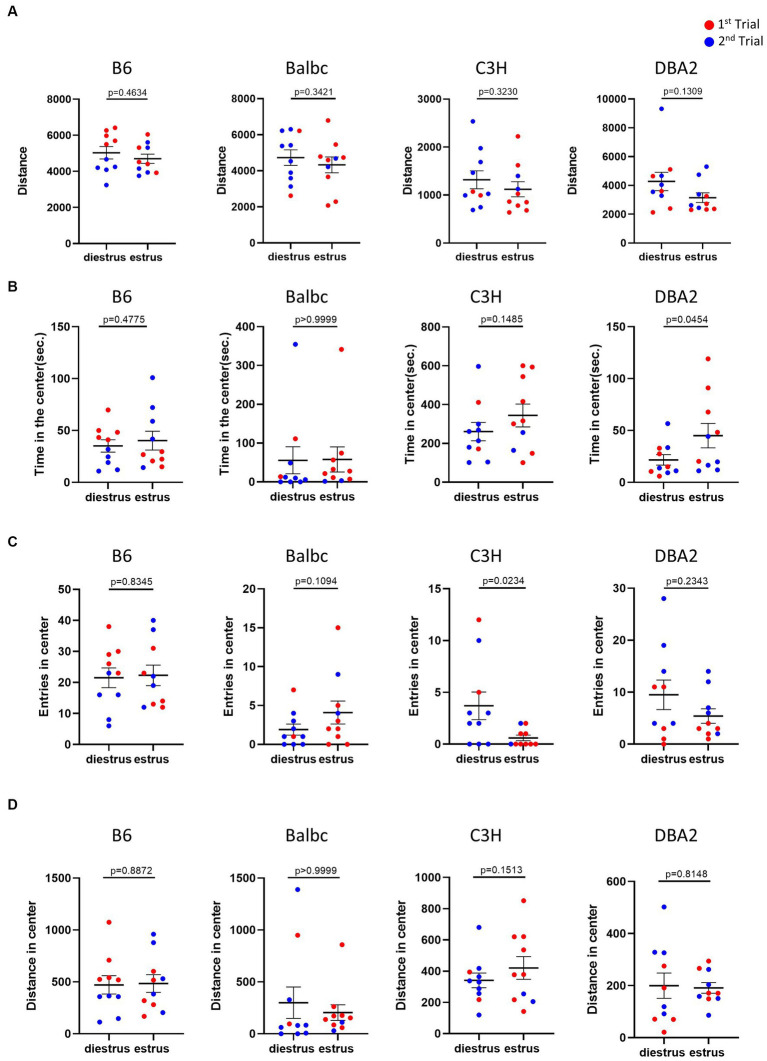
Estrous stage has no significant influence on female mouse activity and anxiety-like behavior, except for time in center in DBA2 and number of entries in C3H. **(A)** The total travel distance of estrus and diestrus female mice in the open-field test. **(B)** The time of estrus and diestrus female mice in the center area of the open-field test. **(C)** The number of estrus and diestrus female mice entered the center area of the open-field test. **(D)** The distance of estrus and diestrus female mice in the center area of the open-field test. Red and blue indicate the first and the second time, respectively, that mice were run in the behavioral assays. Paired *t*-test or Wilcoxon matched-pairs signed-rank test, Mean ± S.E.M. (n = 10 for each group).

The open field test is commonly used to monitor anxiety-like behavior and locomotion in mice. In the open field test, for all four strains, the results showed no significant difference in the overall locomotor activity, indicated by the total distance traveled, between estrus and diestrus females (Figure 2A). The anxiety-like behavior reflected by the time spent, entries and distance in the center of the open field test arena was also not affected by the estrous cycle in most parameters among four strains (Figures 2B–D). The only significant differences we detected was more time in the center for DBA2 estrus mice and lower number of entries for estrus C3H.

**Figure 3:**
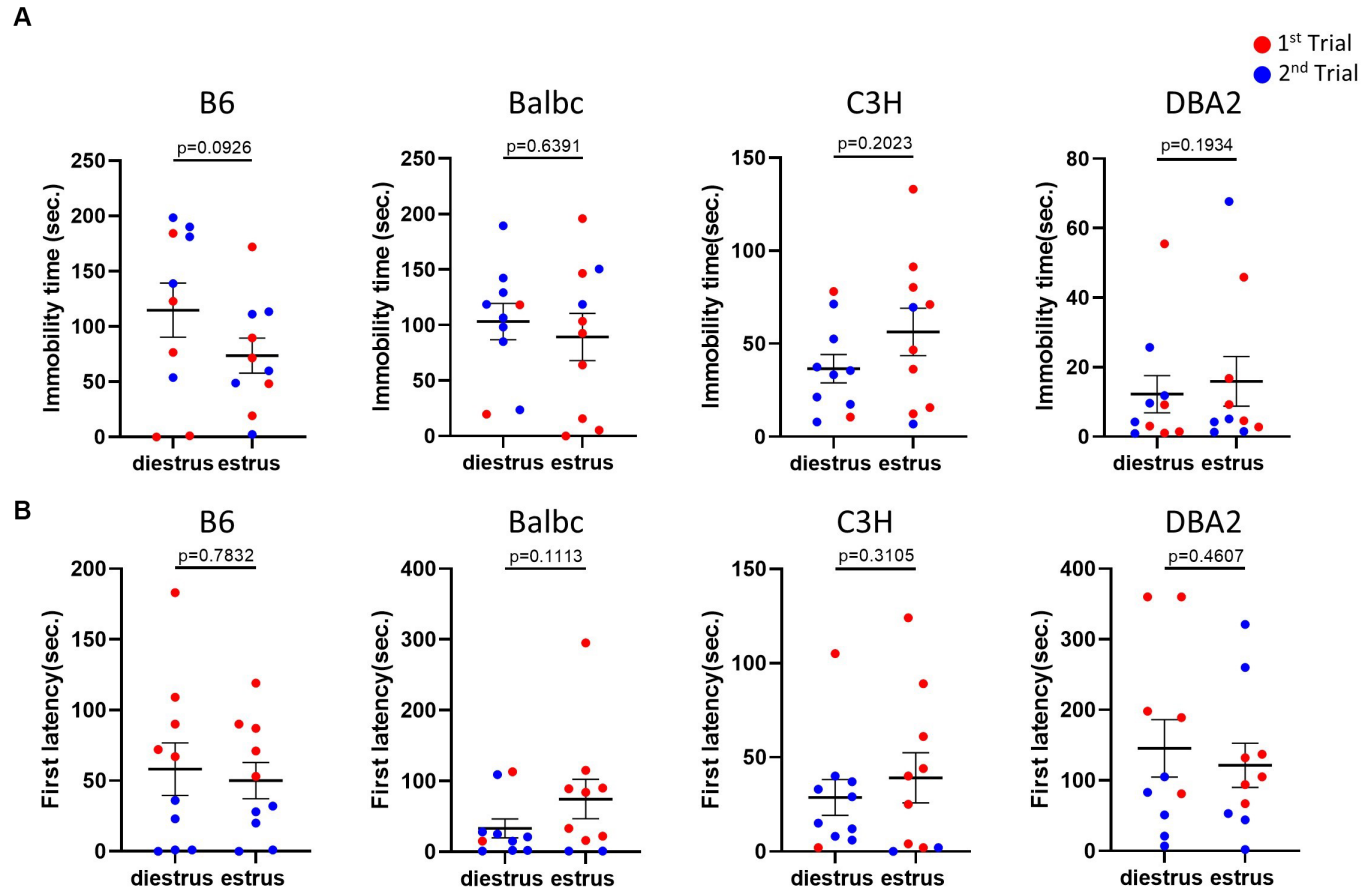
Estrous stage has no significant influence on female mouse depression-like behavior. **(A)** The immobile time of estrus and diestrus female mice in the forced-swimming test. **(B)** The latency to immobility of estrus and diestrus female mice in the forced-swimming test. Red and blue indicate the first and the second time, respectively, that mice were run in the behavioral assays. Paired *t*-test or Wilcoxon matched-pairs signed-rank test, Mean ± S.E.M. (*n* = 10 for each group).

Next, we used the forced swimming test to evaluate the influence of the estrous stage on depression-like behavior. In all strains, females in the estrus and diestrus stage were not different in the immobile time and the latency to immobility of the forced swimming test (Figures 3A,B), suggesting no significant effect of the estrous cycle on depression-like behavior.

**Figure 4:**
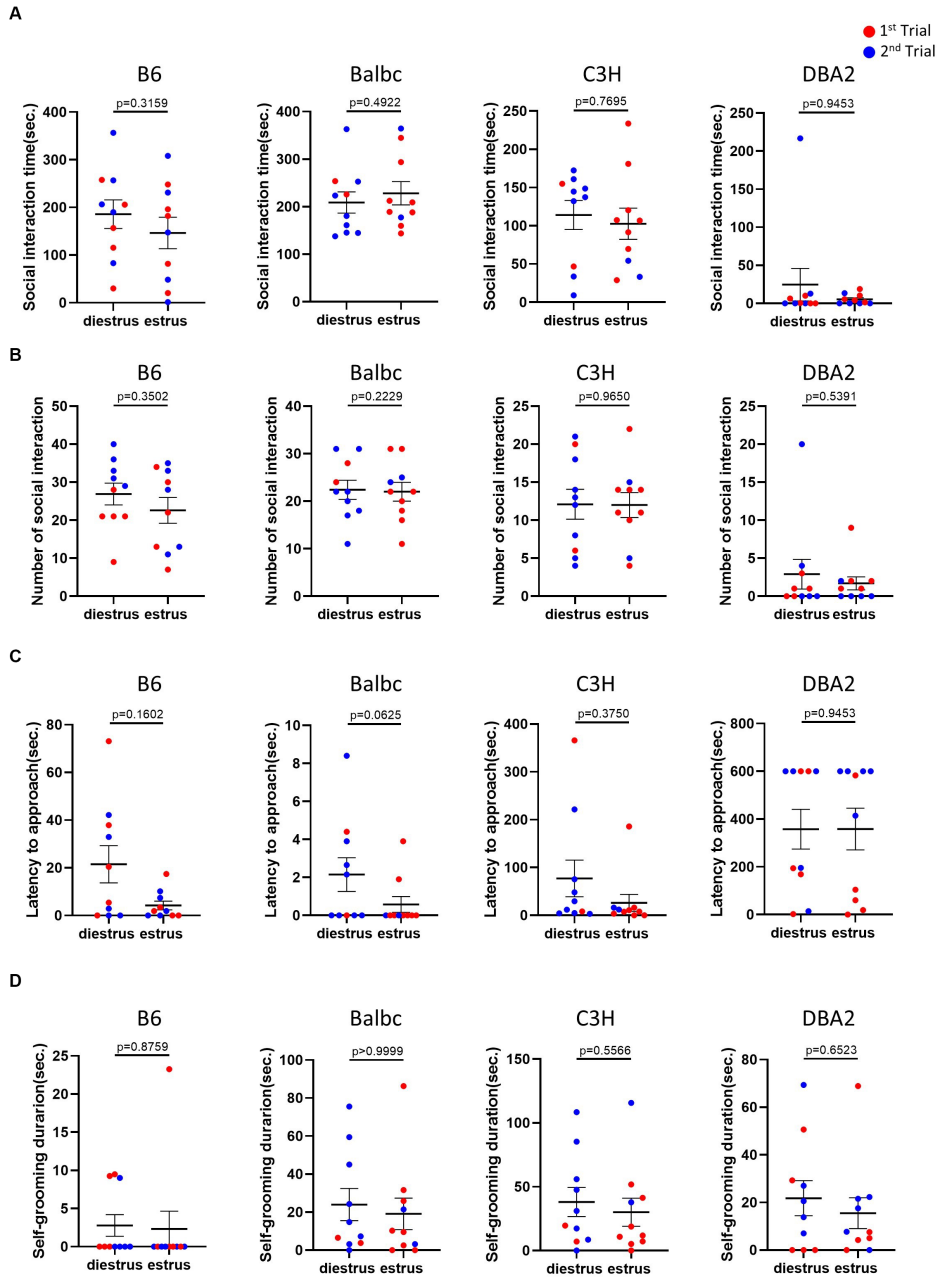
Estrous stage has no significant influence on female mouse social and self-grooming behaviors. **(A)** The total social interaction time of estrus and diestrus female mice with intruders in the resident-intruder assay. **(B)** The number of social bouts of estrus and diestrus female mice in the resident-intruder assay. **(C)** The latency of the estrus and diestrus female approaching the intruder in the resident-intruder assay. **(D)** The self-grooming time of estrus and diestrus female mice in the resident-intruder assay. Red and blue indicate the first and the second time, respectively, that mice were run in the behavioral assays. Paired *t*-test or Wilcoxon matched-pairs signed-rank test, Mean ± S.E.M. (*n* = 10 for each group).

In the resident-intruder assay, we examined whether female mice in different estrous stages exhibited distinct social motivation with another female intruders. Behavioral analysis showed no significant difference in social interaction time, number of social bouts and latency to approach between females in the estrus and diestrus stages (Figures 4A–C). We also examined self-grooming behavior, which can potentially reflect mouse anxiety level (Kalueff et al., 2016), during the intruder assay but detect no difference (Figure 4D).

Given that each mouse was tested twice in our experiments, once during the estrus stage and once during the diestrus stage in a randomized order, it is possible that the repeated tests could impact the behavioral performance of the mice. However, for C57BL/6 and DBA2, there happened to be 5 estrus and 5 diestrus mice tested in the 1st trial (and the 2nd trial), which counterbalanced the potential influence of repeated behavioral tests. To further account for this potential trial effect, we utilized GLMM to assess the behavioral differences between estrus and diestrus females, with strain considered as a random effect (Table 1). While there was a significant trial effect on the latency to immobility, the stages themselves as well as the interactions between stage and trial did not demonstrate any significant effects on the observed behaviors. Consequently, our findings suggested that the influence of the estrous cycle on the female behaviors we examined is very minimal.

**Table 1:** Results of GLMM for the effects of the stage and trial on female behaviors.

		Coefficient	SE	*t* value	*p*-value
**Travel distance**	**Intercept**	8.041	0.334	24.063	**<0.001** ^*^
	**Stage**	0.108	0.121	0.889	0.377
	**Trial**	−0.1	0.121	−0.823	0.413
	**Interaction**	0.056	0.176	0.317	0.752
**Time in center**	**Intercept**	4.046	0.561	7.207	**<0.001** ^*^
	**Stage**	0.046	0.361	0.127	0.899
	**Trial**	0.455	0.358	1.271	0.208
	**Interaction**	−0.387	0.52	−0.744	0.459
**Entries in center**	**Intercept**	2.143	0.449	4.774	**<0.001** ^*^
	**Stage**	−0.156	0.307	−0.509	0.613
	**Trial**	−0.513	0.309	−1.661	0.102
	**Interaction**	0.765	0.438	1.744	0.086
**Distance in center**	**Intercept**	5.638	0.279	20.236	**<0.001** ^*^
	**Stage**	0.109	0.279	0.391	0.679
	**Time**	0.12	0.276	0.434	0.666
	**Interaction**	0.019	0.399	0.047	0.962
**Immobility time**	**Intercept**	3.857	0.498	7.74	**<0.001** ^*^
	**Stage**	0.099	0.313	0.316	0.753
	**Trial**	0.078	0.313	0.248	0.804
	**Interaction**	−0.276	0.453	−0.608	0.545
**Latency to immobility**	**Intercept**	3.601	0.387	9.316	**<0.001** ^*^
	**Stage**	−0.269	0.348	−0.772	0.442
	**Trial**	0.832	0.345	2.413	0.018*
	**Interaction**	0.526	0.489	1.075	0.286
**Social interaction time**	**Intercept**	4.425	0.571	7.744	**<0.001** ^*^
	**Stage**	0.533	0.335	1.592	0.116
	**Trial**	0.041	0.329	0.123	0.902
	**Interaction**	−0.775	0.464	−1.672	0.099
**Number of social bouts**	**Intercept**	2.474	0.452	5.467	**<0.001** ^*^
	**Stage**	0.8	0.228	1.265	0.21
	**Trial**	0.001	0.224	0.006	0.995
	**Interaction**	−0.415	0.316	−1.313	0.194
**Latency to approach**	**Intercept**	3.566	1.005	3.549	**0.001** ^*^
	**Stage**	0.053	0.478	0.111	0.912
	**Trial**	−0.59	0.487	−1.212	0.231
	**Interaction**	1.053	0.678	1.554	0.126
**Self-grooming**	**Intercept**	3.431	0.342	10.038	**<0.001** ^*^
	**Stage**	0.166	0.404	0.412	0.682
	**Trial**	−0.277	0.411	−0.673	0.504
	**Interaction**	−0.492	0.577	−0.853	0.398

The strain was designated as a random effect. Bold indicates *p*-value <0.05.

### The behavioral differences among four strains were similar between estrus and diestrus females

**Figure 5:**
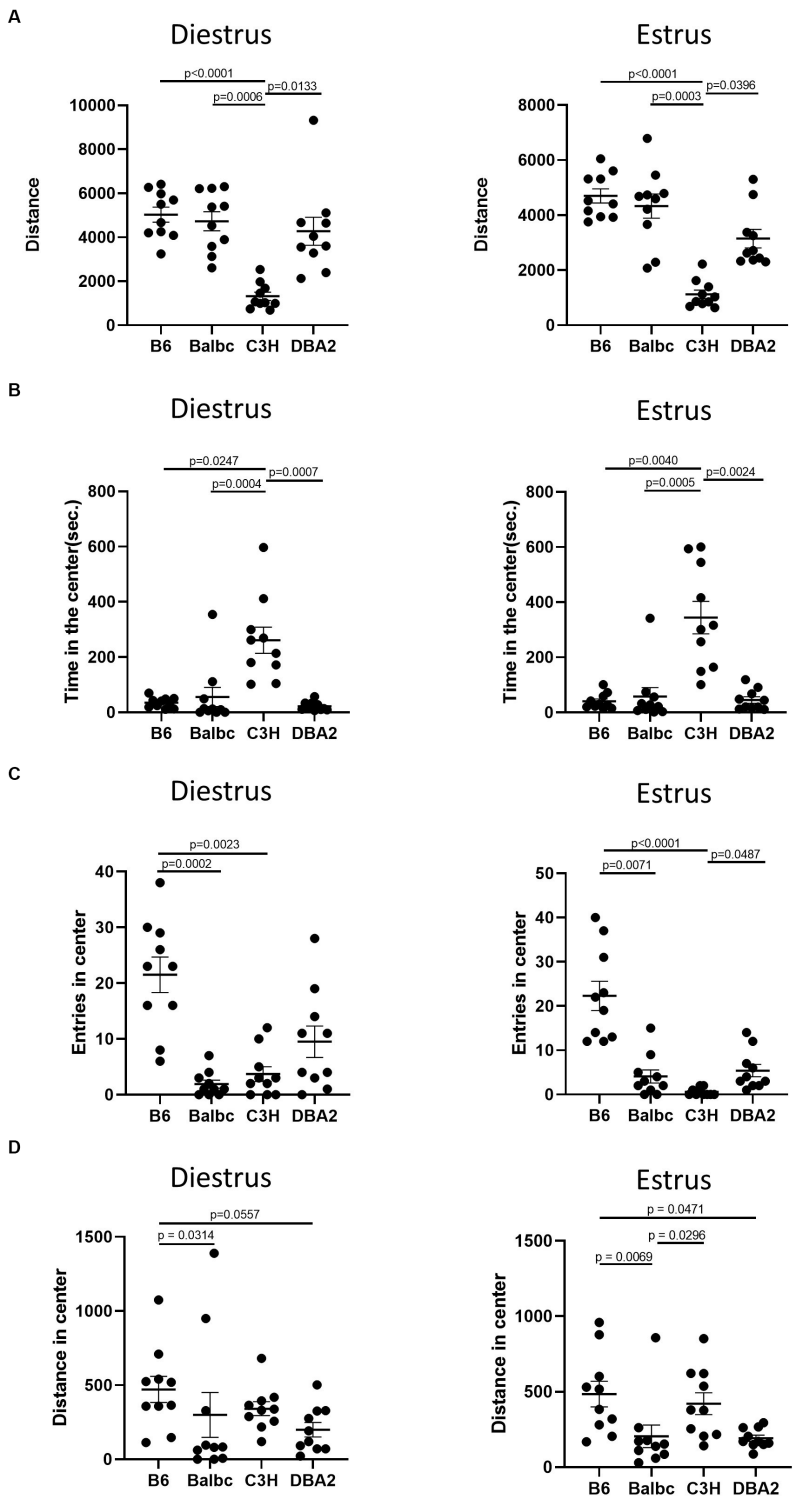
Females showed similar differences among four strains between the estrus and diestrus stage on locomotor and anxiety-like behavior. **(A)** The total travel distance of estrus and diestrus female mice among four different strains in the open-field test (Diestrus: H (3) = 23.15, *p* < 0.0001, Estrus: H (3) = 25.74, *p* = 0.0001). **(B)** The time of estrus and diestrus female mice among four different strains in the center area of the open-field test (Diestrus: H (3) = 20.62, *p* < 0.0001, Estrus: H (3) = 19.96, *p* = 0.0002). **(C)** The number of estrus and diestrus female mice entered the center area of the open-field test (Diestrus: H (3) = 20.89, *p* = 0.0001, Estrus: H (3) = 27.25, *p* < 0.0001). **(D)** The distance of estrus and diestrus female mice in the center area of the open-field test (Diestrus: H (3) = 11.22, *p* = 0.0106, Estrus: H (3) = 15.51, *p* = 0.0014). Kruskal-Wallis test followed by the Dunn’s Multiple comparisons, Mean ± S.E.M. (*n* = 10 for each group).

Although we failed to reveal significant effect of estrous cycle on most female behaviors, multiple differences among four different strains can be easily detected. In the open field test, in both estrus and diestrus females, C3H mice showed less total distance traveled and more time spent in the center than all other strains in both estrus and diestrus females (Figures 5A,B). C57BL/6 in both stages showed more entries in the center than Balbc and C3H (Figure 5C). C3H also tended to have lower number of entries in the center than DBA2, but only significant in estrus stage. These results together suggested that C3H may prefer to stay in the center without moving during the assay. In addition, C57BL/6 in both stages showed more distances in center than BalbC and DBA2 (Figure 5D).

**Figure 6:**
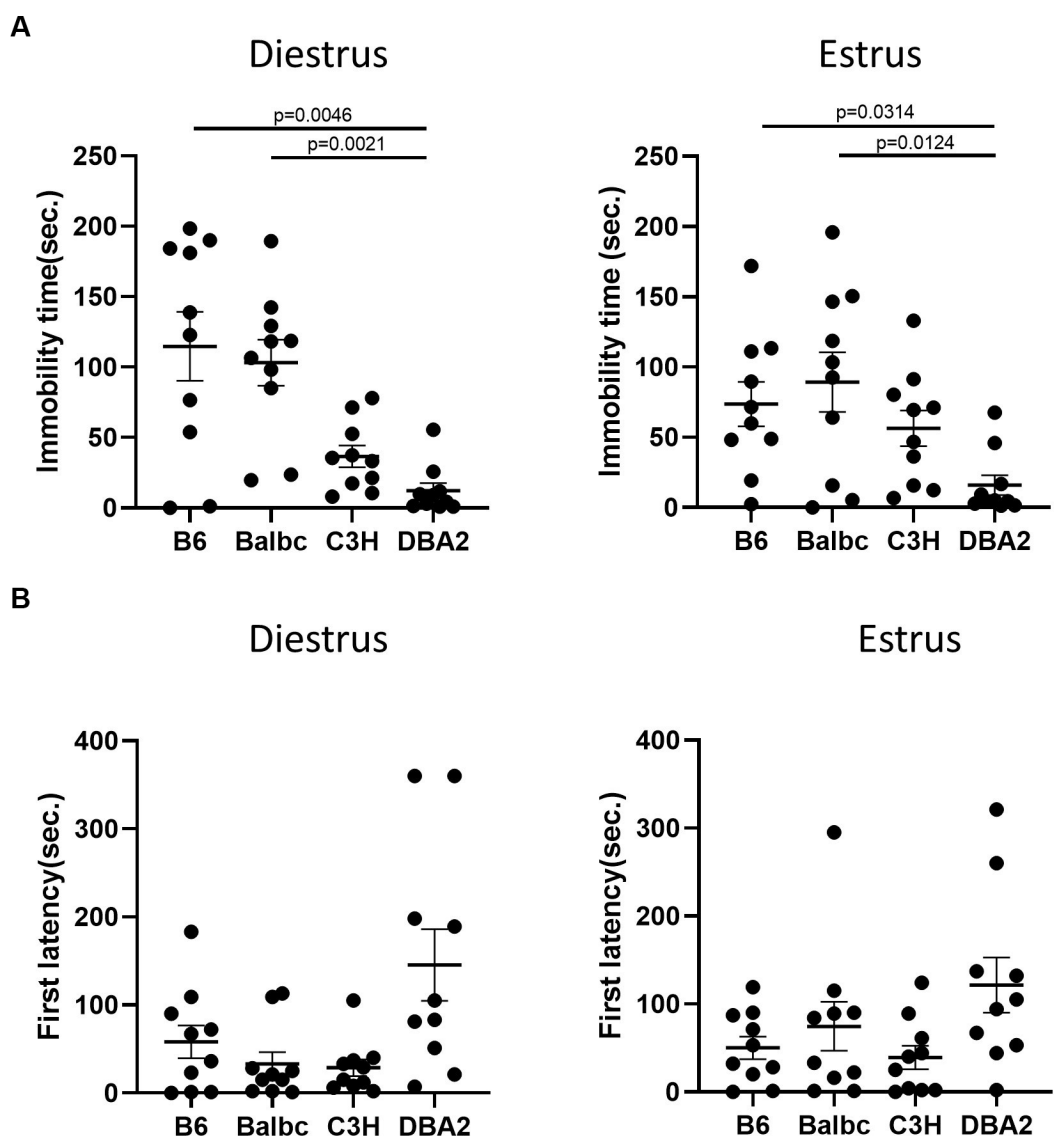
Females showed similar differences among four strains between the estrus and diestrus stages on depression-like behavior. **(A)** The immobile time of estrus and diestrus female mice among four different strains in the forced-swimming test (Diestrus: H (3) = 16.99, *p* = 0.0007, Estrus: H (3) = 11.69, *p* = 0.0085). **(B)** The latency to immobility of the estrus and diestrus female mice among four different strains in the forced-swimming test (Diestrus: H (3) = 7.963, *p* = 0.0468, Estrus: H (3) = 6.605, *p* = 0.0856). Kruskal-Wallis test followed by the Dunn’s Multiple comparisons, Mean ± S.E.M. (*n* = 10 for each group).

In the forced swimming test, DBA2 females in both stages showed shorter immobile time than C57BL/6 and Balbc females (Figure 6A). DBA2 also tended to have more latency time to first immobility in both stages (Figure 6B), although the data was not significant.

**Figure 7:**
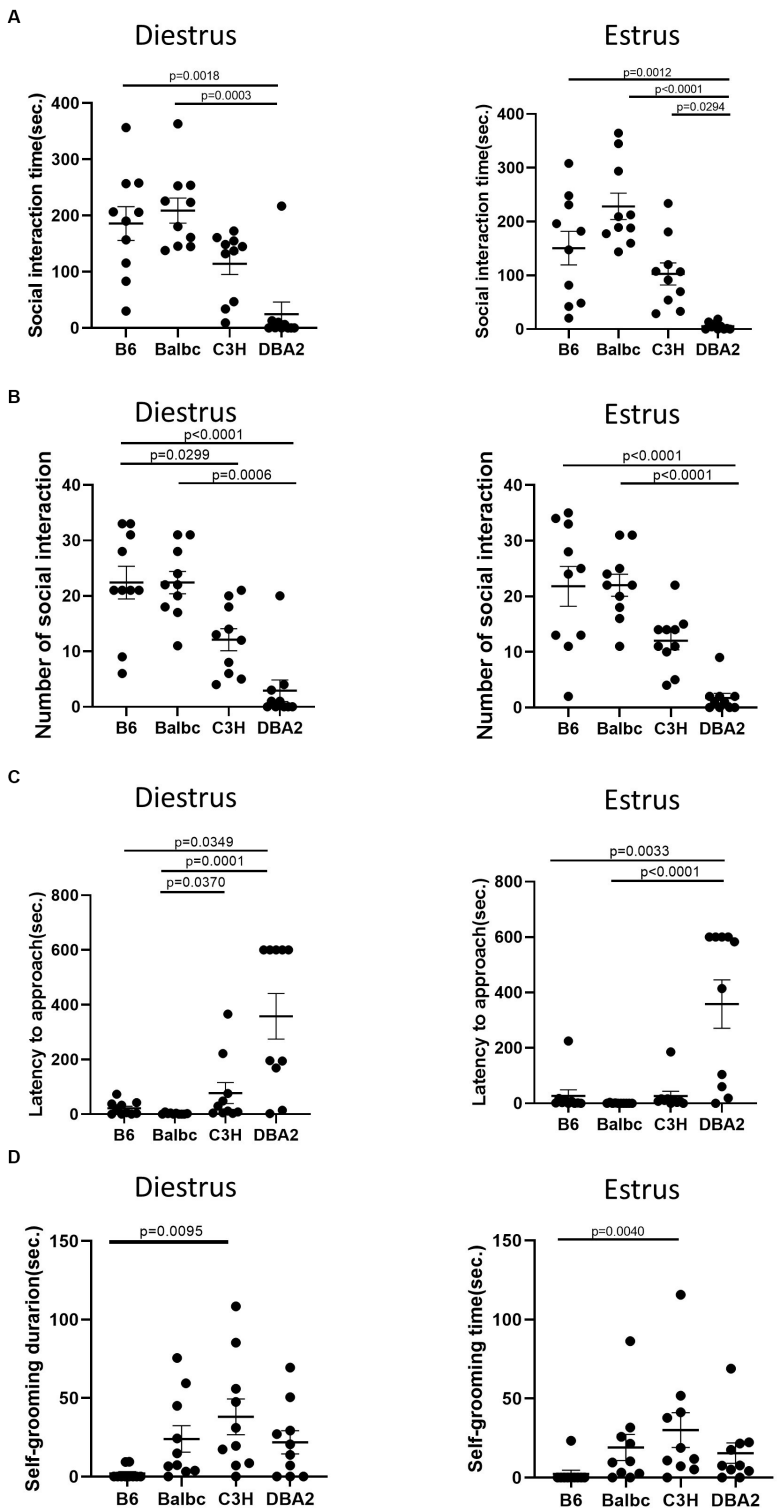
Females showed similar differences among four strains between the estrus and diestrus stage on social behavior. **(A)** The social interaction time of estrus and diestrus female mice among four different strains in the resident-intruder assay (Diestrus: H (3) = 19.66, *p* = 0.0002, Estrus: H (3) = 25.32, *p* < 0.0001). **(B)** The number of social bouts of estrus and diestrus female mice among four different strains in the resident-intruder assay (Diestrus: H (3) = 24.33, *p* < 0.0001, Estrus: H (3) = 25.36, *p* < 0.0001). **(C)** The latency of the estrus and diestrus female mice approaching the intruder among four different strains in the resident-intruder assay (Diestrus: H (3) = 20.69, *p* = 0.0001, Estrus: H (3) = 22.47, *p* < 0.0001). **(D)** The self-grooming time of estrus and diestrus female mice among four different strains in the resident-intruder assay (Diestrus: H (3) = 12.68, *p* = 0.0054, Estrus: H (3) = 12.65, *p* = 0.0055). Kruskal-Wallis test followed by the Dunn’s Multiple comparisons, Mean ± S.E.M. (*n* = 10 for each group).

In the resident-intruder assay, again for both estrus and diestrus females, DBA2 females showed less social interaction time and fewer number of social bouts than C57BL/6 and BalbC (Figures 7A,B). DBA2 showed longer latency than C57BL/6 and BalbC (Figure 7C), and C3H showed more self-grooming than C57BL/6 in both stages (Figure 7D).

**Figure 8:**
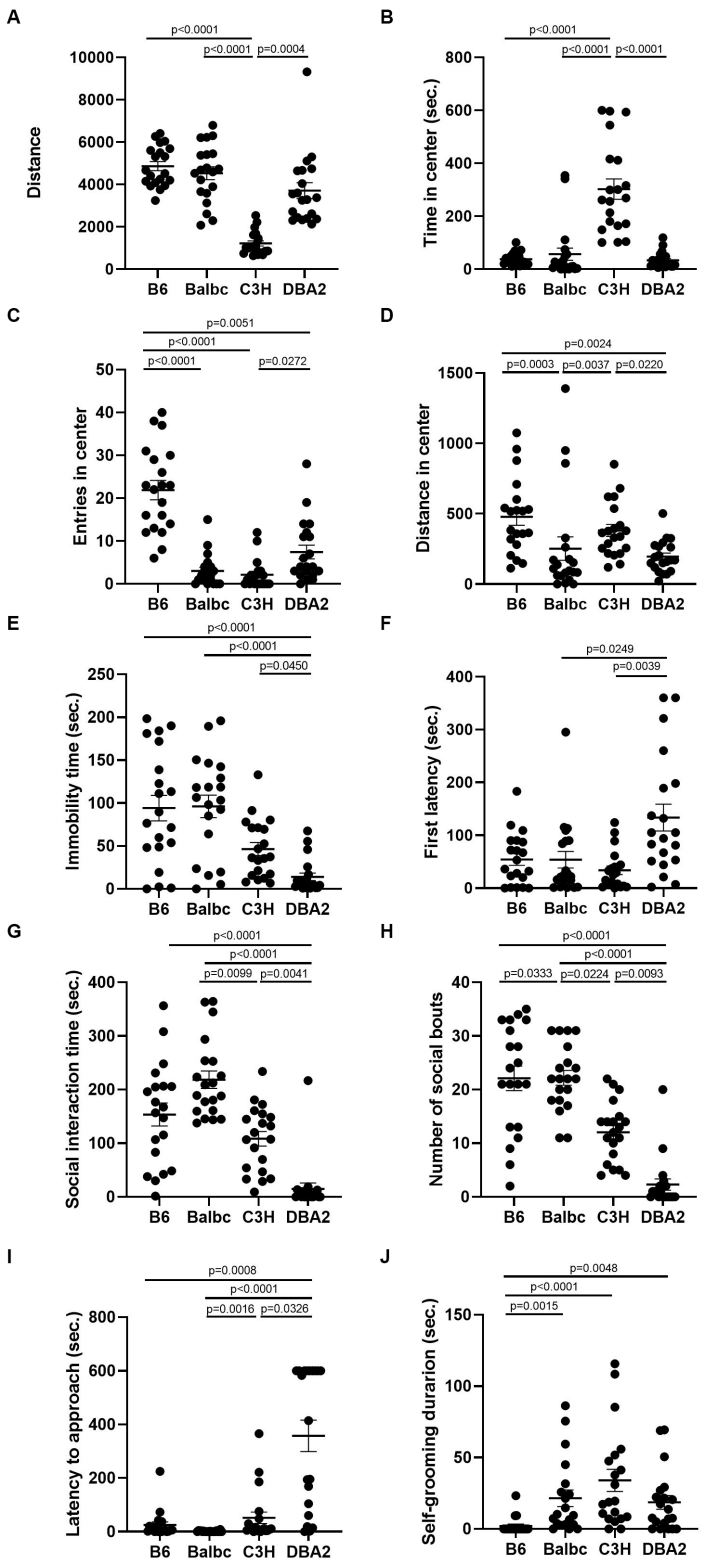
Combining female data from both estrus and diestrus stages revealed many behavioral differences among the four strains. **(A)** The total travel distance of female mice among four different strains in the open-field test (H (3) = 48.75, *p* < 0.0001). **(B)** The time of female mice among four different strains in the center area of the open-field test (H (3) = 40.78, *p* < 0.0001). **(C)** The number of female mice among four different strains entered the center area of the open-field test (H (3) = 45.46, *p* < 0.0001). **(D)** The distance of female mice among four different strains in the center area of the open-field test (Diestrus: H (3) = 24.95, *p* < 0.0001). **(E)** The immobile time of female mice among four different strains in the forced-swimming test (H (3) = 28.39, *p* < 0.0001). **(F)** The latency to immobility of female mice among four different strains in the forced-swimming test (H (3) = 13.76, *p* = 0.0033). **(G)** The social interaction time of female mice among four different strains in the resident-intruder assay (H (3) = 46.00, *p* < 0.0001). **(H)** The number of social bouts of female mice among four different strains in the resident-intruder assay (H (3) = 42.47, *p* < 0.0001). **(I)** The latency of the female mice approaching the intruder among four different strains in the resident-intruder assay (H (3) = 49.10, *p* < 0.0001). **(J)** The self-grooming time of female mice among four different strains in the resident-intruder assay (H (3) = 25.65, *p* < 0.0001). Kruskal-Wallis test followed by Dunn’s Multiple comparisons, Mean ± S.E.M. (*n* = 20 for each group, 10 mice tested in both estrus and diestrus stages).

Again, to account for the trial effect, we employed a GLMM with stage designated as a random effect to evaluate the influence of strain on the behavioral parameters, with C57BL/6 designated as the baseline. The results revealed significant strain effects on almost all parameters (except for Distance in center and Self-grooming), while the trial effect was only significant in the latency to immobility (Table 2). Furthermore, significant interactions between trial and strain were observed in certain parameters (Travel distance, Entries in center, Social interaction time and Number of social bouts). Overall, while the estrous cycle exhibited negligible influence on most female behaviors, our study successfully identified multiple distinctions among the strains. Notably, many of these differences were highly consistent between females in both estrus and diestrus stages. By combining data from both stages and doubling the sample size, additional disparities among the four strains can be further elucidated (Figure 8).

**Table 2:** Results of GLMM for the effects of the strain and trial on female behaviors.

		Coefficient	SE	*t* value	*p*-value
**Travel distance**	**Intercept**	8.374	0.117	71.413	**<0.001** ^*^
	**BalbC**	0.068	0.154	0.444	0.658
	**C3H**	−1.176	0.154	−7.64	**<0.001** ^*^
	**DBA2**	−0.004	0.153	−0.025	0.98
	**Trial**	0.218	0.153	1.429	0.157
	**DBA2** ^*^ **Trial**	−0.559	0.216	−2.585	**0.012** ^*^
**Time in center**	**Intercept**	3.656	0.334	10.958	**<0.001** ^*^
	**BalbC**	0.343	0.5	0.686	0.495
	**C3H**	1.844	0.472	3.908	**<0.001** ^*^
	**DBA2**	−0.527	0.472	−1.116	0.268
	**Trial**	−0.053	0.472	−0.115	0.911
**Entries in center**	**Intercept**	2.998	0.225	11.755	**<0.001** ^*^
	**Balbc**	−1.764	0.389	−4.53	**<0.001** ^*^
	**C3H**	−1.719	0.409	−4.206	**<0.001** ^*^
	**DBA2**	−0.606	0.353	−1.718	0.091
	**Trial**	0.17	0.353	0.483	0.631
	**DBA2** ^*^ **Trial**	−1.104	0.506	−2.182	0.033*
**Distance in center**	**Intercept**	6.603	0.266	22.776	**<0.001** ^*^
	**BalbC**	−0.497	0.399	−1.246	0.217
	**C3H**	−0.277	0.376	−0.736	0.464
	**DBA2**	−0.656	0.376	−1.742	0.086
	**Trial**	0.202	0.376	0.535	0.594
**Immobility time**	**Intercept**	4.699	0.297	15.842	**<0.001** ^*^
	**BalbC**	−1.134	0.419	0.136	0.892
	**C3H**	−1.134	0.419	−2.704	0.009*
	**DBA2**	−2.118	0.419	−5.049	**<0.001** ^*^
	**Trial**	−0.335	0.419	−0.798	0.428
**Latency to immobility**	**Intercept**	2.876	0.341	8.427	**<0.001** ^*^
	**BalbC**	1.674	0.458	0.315	0.754
	**C3H**	0.13	0.469	0.278	0.782
	**DBA2**	1.674	0.458	3.656	**<0.001** ^*^
	**Trial**	1.668	0.458	3.642	**<0.001** ^*^
**Social interaction time**	**Intercept**	5.321	0.189	28.135	**<0.001** ^*^
	**BalbC**	0.05	0.267	0.188	0.851
	**C3H**	−0.689	0.267	−2.578	**0.012** ^*^
	**DBA2**	−0.926	0.394	−2.353	**0.022** ^*^
	**Trial**	−0.438	0.267	−1.637	0.107
	**DBA2** ^*^ **Trial**	−1.997	0.485	−4.116	**<0.001** ^*^
**Number of social bouts**	**Intercept**	3.364	0.174	19.328	**<0.001** ^*^
	**BalbC**	−0.268	0.246	−1.09	0.28
	**C3H**	−0.921	0.246	−3.744	**<0.001** ^*^
	**DBA2**	−1.204	0.362	−3.324	**<0.001** ^*^
	**Trial**	−0.339	0.246	−1.376	0.174
	**DBA2** ^*^ **Trial**	−0.905	0.447	−2.206	**0.047** ^*^
**Latency to approach**	**Intercept**	2.614	0.612	4.27	**<0.001** ^*^
	**BalbC**	−1.537	0.741	−2.075	**0.043** ^*^
	**C3H**	0.837	0.587	1.425	0.16
	**DBA2**	3.692	0.58	6.364	**<0.001** ^*^
	**Trial**	0.285	0.625	0.455	0.651
**Self-grooming**	**Intercept**	2.199	0.914	2.405	**0.020** ^*^
	**BalbC**	1.143	0.964	1.186	0.242
	**C3H**	1.834	0.964	1.903	0.063
	**DBA2**	0.937	0.964	0.972	0.336
	**Trial**	0.441	1.056	0.418	0.678

The stage was designated as a random effect. BalbC, C3H and DBA2 were compared to B6, which was assigned as the baseline. For interaction between strain and trial, only interactions with significance were listed. Bold indicates *p*-value <0.05.

### Similar behavioral variation between males and females

**Figure 9:**
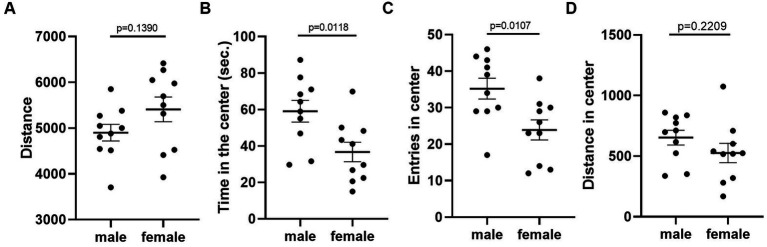
C57BL/6 males showed less anxiety-like behavior than females. **(A)** The total travel distance of male and female mice in the open-field test. **(B)** The time of male and female mice in the center area of the open-field test. **(C)** The number of male and female mice entered the center area of the open-field test. **(D)** The distance of male and female mice in the center area of the open-field test. Welch’s t-test, Mean ± S.E.M. (*n* = 10 for each group).

**Figure 10:**
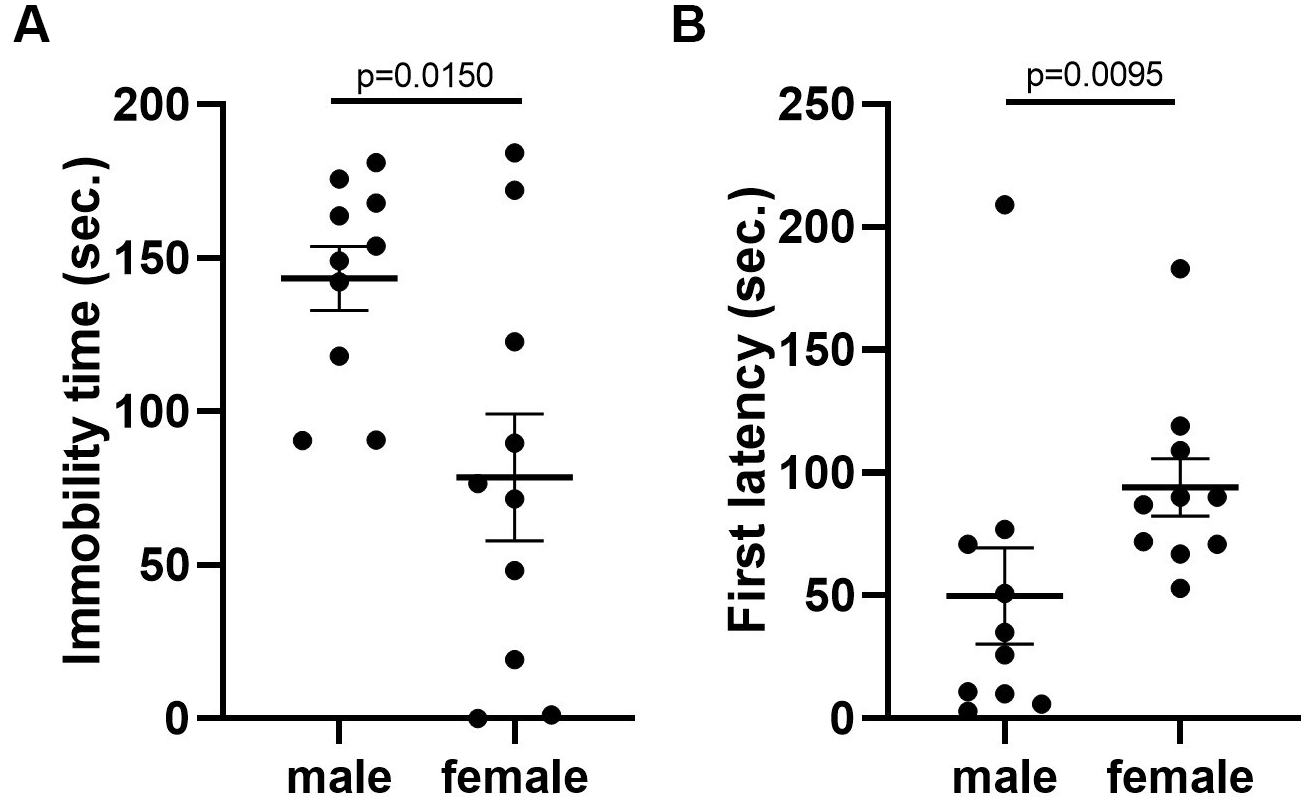
C57BL/6 males showed higher depression-like behavior than females. **(A)** The immobile time of male and female mice in the forced-swimming test. Welch’s *t*-test. **(B)** The latency to immobility of male and female mice in the forced-swimming test. Mann–Whitney *U* test. Mean ± S.E.M. (*n* = 10 for each group).

**Figure 11:**
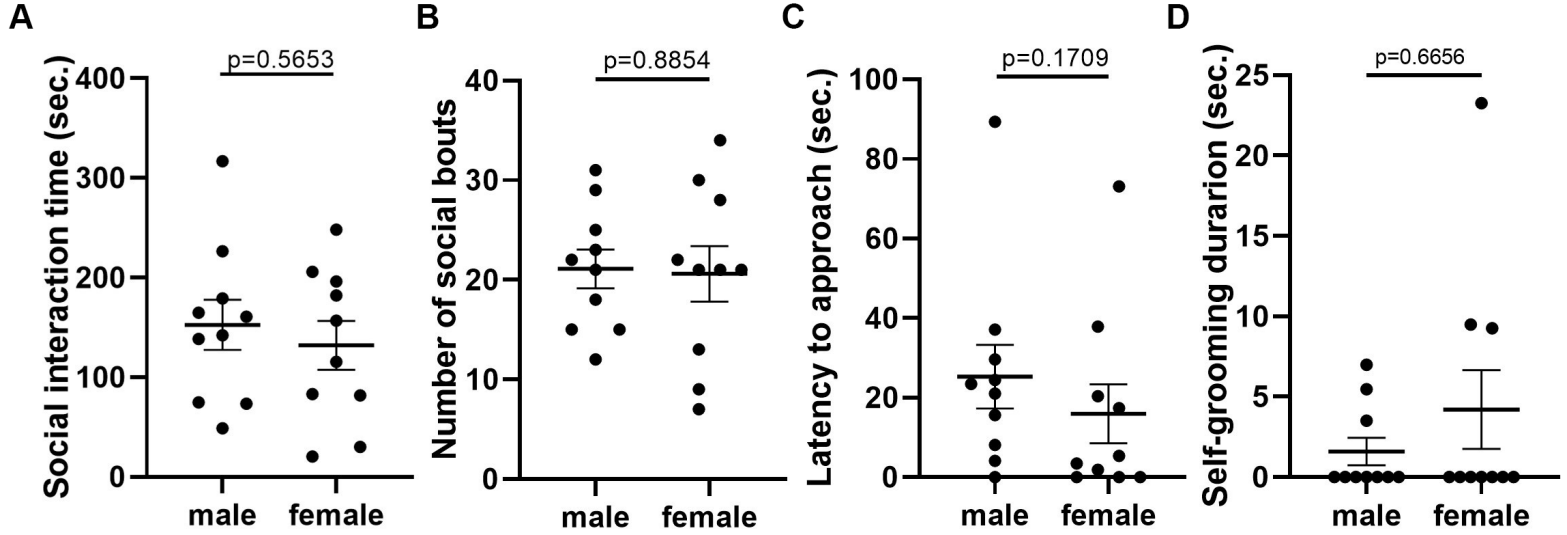
There was no significant difference in social and self-grooming behaviors between C57BL/6 male and female mice. **(A)** The total social interaction time of male and female mice with same-sex intruders in the resident-intruder assay. Welch’s *t*-test. **(B)** The number of social bouts of male and female mice in the resident-intruder assay. Mann–Whitney *U* test. **(C)** The latency of the male and female resident approaching the intruder in the resident-intruder assay. Welch’s *t*-test. **(D)** The self-grooming time of male and female mice in the resident-intruder assay. Mann–Whitney *U* test. Mean ± S.E.M. (*n* = 10 for each group).

Given that the estrous cycle did not cause significant change in behaviors of female mice, we speculated whether the behavioral variation in females is significantly larger or different from male mice. We therefore conducted the same experiments in C57BL/6 male mice. We first examined the behavioral difference between male and female mice, using the data from females tested in the first trial. The results showed that there was no difference between male and female in locomotor activity (Figure 9A). However, we observed more anxiety-like behavior in female mice, reflected by less time spent and less entries in the center of the open-field arena (Figures 9B,C). The distance in center was not different between the two sexes (Figure 9D). In the forced swimming test, males exhibited longer immobile time and shorter latency to immobile, indicating higher depression-like level (Figures 10A,B). In the resident-intruder assay, male and female mice showed similar time in social interaction and self-grooming (Figures 11A–D).

To compare the variation between male and female behaviors, we aggregated the data from females in both stages and calculated the standard deviation (SD) and coefficient of variation (CoV). For males, we repeated the behavioral assays and combined the data from both trials to calculate the SD and CoV. For SD, which can reflect the degree of variation among individuals within a group, we observed higher values in females for most tested behaviors but not entries in the center, social interaction time and latency to approach (Table 3). For CoV, which stands for the extent of variability in relation to the mean of the population, females showed higher values in parameters for open-field test but lower values in parameters for forced swimming test. In addition, the CoV values of female was higher for the number of social bouts, latency to approach and self-grooming, but lower for social interaction time (Table 3). Even though females showed higher SD and CoV in some assays, Levene’s test suggested that the inequality between male and female variation was statistically significant only in the locomotor activity and the number of social bouts (Table 3). F-test, which can be used to examine normally distributed data, showed that the difference was statistically significant only in the number of social bouts. In summary, for most of the studied behaviors, males and females showed very similar variability. Females showed larger variation only in the locomotor activity and the number of social bouts.

**Table 3:** The variations of behaviors in male and female mice.

	SD		CoV		Levene	*F*-test
	Male	Female	Male	Female	–	–
Travel distance	732.104	944.219	0.154	0.194	0.045*	0.276
Time in center	21.003	23.707	0.457	0.629	0.745	–
Entries in center	10.589	9.989	0.357	0.456	0.900	0.802
Distance in center	215.711	267.246	0.391	0.560	0.383	0.359
Immobility time	44.707	66.747	0.709	0.335	0.468	0.089
Latency to immobility	48.792	49.319	1.640	0.911	0.433	–
Social interaction time	136.199	95.903	0.663	0.570	0.127	–
Number of social bouts	5.917	9.920	0.285	0.401	0.009*	0.030*
Latency to approach	20.500	19.569	1.175	1.523	0.817	–
Self-grooming	4.119	5.929	2.129	2.324	0.305	–

SD, standard deviation. CV, coefficient of variation. *n* = 20 for each group (10 mice with one replication). Bold indicates *p*-value <0.05.

## Discussion

It is generally believed that hormone change by estrous cycle would not only affect sexual behaviors but also cause large variations in other behavioral assays. However, in this study, we examined anxiety-like, depression-like behaviors and social interaction in multiple inbred strains but found very few behavioral parameters with significant difference between estrus and diestrus females. The differences among four strains were therefore similar in these two stages. Our result also suggested similar behavioral variation between male and female mice in most behavioral traits, except for the locomotor activity and number of social bouts. Together, we found no solid evidence to conclude larger behavioral variation in female mice due to the estrous cycle.

Male and female are different in a variety of phenotypes, from morphology, physiology to behaviors. Our data suggested that male and female are different in anxiety- and depression-like behaviors. Previous reports also showed that individual housing could increase anxiety levels in female mice (Palanza et al., 2001; Takahashi et al., 2017), but not in males (Palanza et al., 2001a; Lander et al., 2017; Heck et al., 2020; Mudra Rakshasa and Tong, 2020; Zilkha et al., 2021). Some previous reports even suggested different circuits to regulate the same behavior between male and female (Taiji et al., 1985; Bangasser and Cuarenta, 2021). Sex differences also manifest in preclinical study in human. For example, the pathogenesis of neurodegenerative diseases is different between the two sexes and influenced by sex hormones (Vegeto et al., 2020). Estrogens were neuroprotective in several neurodegenerative disease (Ji et al., 2017; Vegeto et al., 2020), while androgens may cause some adverse effects in Amyotrophic Lateral Sclerosis (Miller and Warnick, 1989). Given that male and female can be different in so many aspects, both sexes should be always considered in the biomedical experiments.

Female mice have frequently been excluded in biological research due to the estrous cycle (Beery and Zucker, 2011; Will et al., 2017; Chen et al., 2018; Yang et al., 2019; Chevalier et al., 2020; Pati et al., 2020; Zhang et al., 2020; Felippe et al., 2021; Mahadevia et al., 2021; Wu et al., 2021; Zhang et al., 2022). It is often believed that females must be tested at each stage of the estrous cycle to generate reliable data. However, while some studies showed different behaviors at distinct estrous stages (Gangitano et al., 2009; Koonce et al., 2012; Rocks et al., 2022), many studies failed to identify the effect of estrous cycle on female non-sexual behaviors (Chari et al., 2020; Zhao et al., 2021; Francois et al., 2022). We therefore revisited this question in this study but found no significant difference in multiple behavioral assays between estrus and diestrus females in four different strains. Multiple factors or reasons can explain the failure to detect the effect. For example, in order to use software to analyze behaviors, our open field and forced swimming tests were conducted under the light condition, which is known to induce anxiety in mice (Trainor et al., 2006; Miller et al., 2021). In addition, we conducted three behavioral tests in one day with only 90-min breaks between each assay, which can probably induce a lot of stress. These limitations of our experimental design could potentially affect mouse behavioral performance and minimize the difference between the two stages. Nevertheless, the anxiety level (indicated by the time in the center) and the depression level (indicated by the immobility time) of mice in our study were not particularly higher than the levels in many other reports (Can et al., 2012; Koonce et al., 2012; Zhao et al., 2021; Rocks et al., 2022), suggesting that the stress induced by our experiments was probably still within an acceptable range.

Furthermore, even though we did not detect the influence of estrous cycle on female behaviors, our data still showed some behavioral differences in certain strains. For example, C3H tended to have lower activity and more time in the center of open field box than the other three strains. DBA showed lower immobility time and less social interaction than C57BL/6 and Balbc. More importantly, these behavioral differences were quite consistent between estrus and diestrus stages. In addition, our experiments also revealed distinct behaviors between female and male mice. These results suggested that our experimental set-up can successfully detect certain degree of difference. While we cannot completely exclude the impact of estrous cycle, we believed that the difference between estrus and diestrus female is very minimal, at least much smaller than the difference among strains or between the sexes.

Similar to previous reports (Prendergast et al., 2014; Lovick and Zangrossi, 2021; Francois et al., 2022; Rocks et al., 2022), our study found that the behavioral variations in females were generally not greater than those in males. Although some of behaviors showed higher SD and CoV, statistically, there is no significant difference in most parameters according to the Levene test and F-test. In fact, while it is believed larger variation induced by female estrous cycle, a lot of potential factors could also cause variations in males. For example, male mice usually perform more aggression (Trainor et al., 2006), which can potentially result in social hierarchy or social defeat and induce more variation in male behaviors (Horii et al., 2017). In addition, some studies have reported that the infradian cycles of male mice are more variable than females within a single day (Smarr et al., 2017; Smarr and Kriegsfeld, 2022). Recent study based on machine-learning software even suggested larger variation in male open-field behavior (Levy et al., 2023). How do these factors contribute to male behavioral variations reminds to be further studied.

In conclusion, our data cannot well support the assumption that estrous cycle drives larger variation in female behaviors. While our experiments successfully detected the differences among strains and between the sexes, we found very similar behaviors in our testing assays between estrus and diestrus females, suggesting a very minimal impact of estrous cycle on those behaviors. More importantly, our results further showed similar behavioral variation between male and female mice. Together, this study suggested that a prudent approach for future research would be to test both females and males. Where the impact of estrous stage is uncertain, the data can be statistically tested whether female data have a higher variance than male data. Then follow-up studies may be appropriate with staged mice to accounting for estrous cycle influences. Given that a lot of our current knowledge related to behavioral neuroscience is based on male mice, we believed sex-unbiased experimental designs are important to avoid inappropriate generalization of findings between the two sexes, and would be critical for the transparency and reproducibility in both basic and translational research.

## Data availability statement

The original contributions presented in the study are included in the article/supplementary material, further inquiries can be directed to the corresponding author.

## Ethics statement

The animal study was reviewed and approved by the Institutional Animal Care and Use Committee of National Tsing Hua University.

## Author contributions

P-YZ and T-HK designed the experiments and wrote the manuscript. P-YZ, Y-HT, C-LL, and Y-KM performed the experiments. P-YZ and C-LL analyzed the data. All authors contributed to the article and approved the submitted version.

## Funding

The work was supported by the National Science and Technology Council (NSTC 112-2636-B-007-007 Young Scholar Fellowship to T-HK), as well as the Higher Education Sprout Project funded by the Ministry of Education and Ministry of Science and Technology.

## Conflict of interest

The authors declare that the research was conducted in the absence of any commercial or financial relationships that could be construed as a potential conflict of interest.

## Publisher’s note

All claims expressed in this article are solely those of the authors and do not necessarily represent those of their affiliated organizations, or those of the publisher, the editors and the reviewers. Any product that may be evaluated in this article, or claim that may be made by its manufacturer, is not guaranteed or endorsed by the publisher.
